# Segmented Linear Regression Modelling of Time-Series of Binary Variables in Healthcare

**DOI:** 10.1155/2019/3478598

**Published:** 2019-12-06

**Authors:** Epaminondas Markos Valsamis, Henry Husband, Gareth Ka-Wai Chan

**Affiliations:** ^1^Nuffield Orthopaedic Centre, Oxford University Hospitals NHS Foundation Trust, Oxford OX3 7LD, UK; ^2^Faculty of Mathematics, University of Cambridge, Cambridge CB3 0WA, UK; ^3^Brighton and Sussex University Hospitals NHS Trust, Brighton BN2 5BE, UK

## Abstract

**Introduction:**

In healthcare, change is usually detected by statistical techniques comparing outcomes before and after an intervention. A common problem faced by researchers is distinguishing change due to secular trends from change due to an intervention. Interrupted time-series analysis has been shown to be effective in describing trends in retrospective time-series and in detecting change, but methods are often biased towards the point of the intervention. Binary outcomes are typically modelled by logistic regression where the log-odds of the binary event is expressed as a function of covariates such as time, making model parameters difficult to interpret. The aim of this study was to present a technique that directly models the probability of binary events to describe change patterns using linear sections.

**Methods:**

We describe a modelling method that fits progressively more complex linear sections to the time-series of binary variables. Model fitting uses maximum likelihood optimisation and models are compared for goodness of fit using Akaike's Information Criterion. The best model describes the most likely change scenario. We applied this modelling technique to evaluate hip fracture patient mortality rate for a total of 2777 patients over a 6-year period, before and after the introduction of a dedicated hip fracture unit (HFU) at a Level 1, Major Trauma Centre.

**Results:**

The proposed modelling technique revealed time-dependent trends that explained how the implementation of the HFU influenced mortality rate in patients sustaining proximal femoral fragility fractures. The technique allowed modelling of the entire time-series without bias to the point of intervention. Modelling the binary variable of interest directly, as opposed to a transformed variable, improved the interpretability of the results.

**Conclusion:**

The proposed segmented linear regression modelling technique using maximum likelihood estimation can be employed to effectively detect trends in time-series of binary variables in retrospective studies.

## 1. Introduction

When randomised controlled trials are not feasible, researchers often employ observational study designs to evaluate the impact of an intervention. Change is usually investigated using statistical analysis that compares preintervention to postintervention data. Typically, statistical methods range from simple group comparisons that ignore temporal trends to more sophisticated interrupted time-series (ITS) analyses [1, 2].

Group comparison is generally considered unreliable as it can be influenced by secular trends which are often too subtle to detect by data inspection alone [3]. By accommodating for temporal trends, ITS enables more reliable conclusions but can suffer from bias because its application is focused on a designated point in time (usually the point of the intervention). Consequently, change unrelated to the intervention can be erroneously credited to the intervention. While statistical variations that aim to rectify these shortcomings do exist, they tend to be too mathematically complex or too arbitrary to be reliable and amenable to healthcare researchers [4].

When the outcome under consideration is a binary event, modelling of the time-series usually involves logistic (logarithm of the odds) regression to ensure that the parameters of the model are mathematically sound. Linear regression of a binary variable may result in predicted probabilities greater than 1 or less than 0. Logistic regression avoids this by finding the logarithm of the odds of the binary event (logit). Despite being mathematically sound, any change in the logit represents a change in the *log odds* rather than the *probability* of the binary event, making the detected change difficult to interpret.

The aim of this study was to attempt to rectify these issues by proposing a novel modelling and model-fitting method which describes change patterns in the time-series of a random binary event without bias to the point of the intervention. The proposed method uses piecewise linear sections and finds the best combination of these sections using a systematic procedure that addresses three important limitations of commonly used change detection techniques:

1. Enforcing a specific model to fit the data: an ideal modelling technique must allow multiple models to be tested to determine which is the best description of the time-series.
2. Modelling the logarithm of the odds of a binary variable: directly modelling the binary variable rather than the logarithm of its odds allows for any temporal variation/change to be expressed as that of the probability of the random event.
3. Bias to the point of the studied intervention: an ideal modelling technique will allow an inflection point between two or more temporal segments at time-points separate to the intervention. This is a significant limitation of the classic ITS technique [5].

The proposed modelling method is applied to a retrospective study investigating the trends in patient mortality following fragility neck of femur fractures at a Level 1 Major Trauma Centre over a period of six years.

## 2. Methods

### 2.1. Segmented Linear Regression to Model Time-Series

We have previously published a modelling technique employing segmented least-squares linear regression to fit a set of progressively more complex models to the time-series of outcome measures in a large retrospective study [5]. The models were either a simple plateau or a single straight line or a combination of these by using adjoining sections.

Adjoining linear segments to model the time-dependence of a variable are known as “splines” and have received considerable attention in scientific literature [6–11]. Several models can be created, each model with a greater number of splines or with a greater degree of freedom, creating a set of nested models of increasing complexity.

In this study, we utilise the same set of progressively more complex segmented linear regression models but employ maximum likelihood regression rather than least-squares regression and discuss its advantages when modelling binary variables.

### 2.2. Models

We model the binary dependent variable *y* (taking values 0 or 1) as a piecewise linear function of the independent variable time *t*. The following four models are proposed to fit the time-series (*t*_*i*_, *y*_*i*_) of the binary data, *i* = 1 to *n*. ${\hat{y}}_{i}$ denotes the modelled values of the probability of the binary event.

(i) “Plateau”: a simple average value
  (1) $$\begin{array}{c} {\hat{y}}_{i}=k,\;\text{ for all }t_{i}. \end{array}$$
This simplest of models assumes that the probability of the event remains unchanged over the study period and uses the average value of the time-series of events to represent its probability.
(ii) “Line”: a single straight line of non-zero gradient
  (2) $$\begin{array}{c} {\hat{y}}_{i}=c+m\cdot t_{i},\;i=1\text{ to }n. \end{array}$$
This model determines two parameters (*y*-axis intercept *c* and gradient *m*) that fit a straight line to the data to describe the influence of time on probability. Constraints must be placed to ensure values predicted by the model within the time range considered are between 0 and 1.
(iii) “Line-plateau/plateau-line”: a straight line joined to a plateau or a plateau joined to a straight line.
Line-plateau:
  (3) $$\begin{array}{c} {\hat{y}}_{i}=mt_{i}+c,\;\text{for }i=1\text{ to }j,\\ {\hat{y}}_{i}=k,\;\text{for }i=j+1\text{ to }n, \end{array}$$
where *k*=*mt*_*i*_ + *c*.
Plateau-line:
  (4) $$\begin{array}{c} {\hat{y}}_{i}=k,\;\text{for }i=1\text{ to }j,\\ {\hat{y}}_{i}=m\left(t_{i}-t_{j}\right)+k,\;\text{for }i=j+1\text{ to }n. \end{array}$$
This model joins a linear section to a plateau at a knot to model the temporal variation of probability. The plateau can precede or follow the linear section. The model is described by three parameters, two of which are the parameters of the straight line (*y*-axis intercept *c* and slope *m*) and the third of which is the time instant *t*_*j*_ that corresponds to the knot. Constraints must be placed to ensure that the model yields values $\hat{y}$ between 0 and 1 over the entire time interval.
(iv) “Line-line”: a straight line joined to another straight line
  (5) $$\begin{array}{c} {\hat{y}}_{i}=m_{1}t_{i}+c,\;\text{for }i=1\text{ to }j,\\ {\hat{y}}_{i}=m_{2}\left(t_{i}-t_{j}\right)+k,\;\text{for }i=1\text{ to }j, \end{array}$$
  where *k*=*m*_1_*t*_*i*_ + *c*.

This model fits two straight line sections which are joined at a knot to model probability. Both sections can have non-zero slopes and are joined at the time instant *t*_*j*_, thus requiring a total of four parameters to describe it. Similarly, constraints must be placed to ensure that values ${\hat{y}}_{i}$ are always between 0 and 1.

### 2.3. Model-Fitting

The parameters (intercepts, slopes, and plateaus) of the proposed set of models are derived to maximise the likelihood of the experimental data and, as such, their values are maximum likelihood estimates (MLE) [12]. The plateau model is derived as the average value of *y* which is also a MLE of the value *k* of equation (1). For the other models we use constrained optimisation algorithms to determine MLE values of the model parameters [13]. Such algorithms are typically available as featured functions or procedures in most current programming and statistical packages.

More specifically, we seek values of the parameters *m* and *c* for model (ii- “Line”), or the parameters *m*, *c* and *j* for model (iii- “Line-Plateau/Plateau-Line”), or the parameters *m*_1_, *m*_2_, *c*, and *j* for the model (iv- “Line-line”) that maximise the likelihood function:

(6) $$\begin{array}{c} L=\underset{i}{\prod }{\hat{y}}_{i}^{y_{i}}{\left(1-{\hat{y}}_{i}\right)}^{\left(1-y_{i}\right)}, \end{array}$$

or, equivalently, that maximise the (natural) logarithm of the likelihood function:

(7) $$\begin{array}{c} \ln\;L=\underset{i}{\sum }y_{i}.{\hat{y}}_{i}+\underset{i}{\sum }\left(1-y_{i}\right)\left(1-{\hat{y}}_{i}\right), \end{array}$$

where *y*_*i*_ is the value of the *i*^th^ binary event and ${\hat{y}}_{i}$ is the probability predicted by the model for time *t*_*i*_.

Practically, we minimise the logarithm of the likelihood (equation (7)) as the likelihood becomes unmanageably small for data sets that feature more than a small number of values.

We used the *fmincon* function in MATLAB®, Mathworks, to implement constrained optimisation to find those values of the parameters of the linear sections that yield the minimum of –ln *L* (which corresponds to the maximum of ln *L* in equation (7)) for each of models (ii), (iii), and (iv) [14]. The need for constrained optimisation is necessary to avoid yielding negative values of *y* or values of *y* exceeding 1 at any point in the time-series.

Finding the MLEs of the parameters in models (iii) and (iv) involves selecting the knot that corresponds to the largest among the MLEs of the parameters for *i* = 1 to *n*. This means splitting the data into two sets, evaluating the MLEs for all possible values of the splitting point *j* and finally selecting *j* as the one that yields the supremum among MLEs.

A detailed description of the constrained optimisation procedure that we used, or its background, is beyond the scope of this work as these are well documented and featured in most public domain programming languages [15].

### 2.4. Selecting the Best-Fitting Model

Once all four models are fitted, the best model must be selected to represent the best description of how the time-series changes over the period studied.

More complex models (those with more parameters and/or more segments) expectedly fit the data better than those with fewer parameters, yielding larger likelihood values [16]. However, we applied the null hypothesis of most modelling methods that states that unless a more complex model fits the data *significantly* better, a simpler model should be preferred. To compare models, we chose to use Akaike's information criterion (AIC) as a measure of the goodness of fit of each model [17]:

(8) $$\begin{array}{c} {\text{AIC}}_{\left(q\right)}=2r_{\left(q\right)}-2\;\ln\left(L_{\left(q\right)}\right), \end{array}$$

where *q* is the model descriptor *q* = (i), (ii), (iii), or (iv), *r*_*q*_ is the number of parameters used by the *q*^th^ model, and ln(*L*_*q*_) is the logarithm of the likelihood of the *q*^th^ model. It is readily deduced that *r*_(i)_ = 1 (plateau), *r*_(ii)_ = 2 (single line), *r*_(iii)_ = 3 (line-plateau or plateau–line), and *r*_(iv)_ = 4 (twin line). The AIC is a compromise between goodness of fit and simplicity and is a widely accepted tool in model selection [17, 18]. The model with the smallest AIC is chosen as the best model to describe the temporal change of the probability of the binary random event.

Although the model with the smallest AIC prevails, the other models need not be discarded. They are compared to the best-fitting model by noting their relative likelihood *RL*_*(q)*_ which is obtained as per Keith and Allison [18]:

(9) $$\begin{array}{c} RL_{\left(q\right)}=e^{\left({\text{AIC}}_{\left(\text{best}\right)}-{\text{AIC}}_{\left(q\right)}\right)/2}. \end{array}$$

The relative likelihood *RL*_(*q*)_ can then be used to determine whether the best model is significantly better than another model (*q*) using typical hypothesis testing criteria with a specific level of significance *p*. For example, if the relative likelihoods *RL*_(*q*)_ < 0.05 for all alternative models (*q*), then this will be sufficient to reject all other models in favour of the best-fitting one at the 5% level. In inferential statistics, this means that the probability of erroneously rejecting the other models is less than 5%.

Finally, the best model is used to describe the time-series whereby it is possible to detect change and reveal secular trends.

### 2.5. Application of the Modelling Technique to Hip Fracture Patient Outcomes

This modelling technique was applied to a time-series of data from a Level 1 Major Trauma Centre in the United Kingdom. As part of a retrospective study, patient survival data were collected from April 2011 to September 2016 for patients sustaining fragility fractures of the proximal femur. In July 2015, on the 1551^st^ day (2179^th^ fracture) of the study, a dedicated hip fracture unit (HFU) was introduced within the trust. Results from this study, including an evaluation on the effectiveness of the introduction of the HFU using segmented least-squares linear regression (without the adaptation for binary variables), have been previously published [5]. Using the modelling technique described in the current study, we reanalysed the same retrospective dataset.

We applied the modelling technique to three time-series: 30-day, 120-day, and 365-day patient mortality. Specifically, our data consisted of two sets of 2851 binary values for 30-day and 120-day mortality and a set of 2494 binary values for 365-day mortality over a period of 1995 days (365-day mortality was monitored up to 12^th^ January 2016 resulting in fewer postintervention data points).

We used basic statistical tests to compare patient mortality before the intervention (pre-HFU) to that following the intervention (post-HFU). Since the pre-HFU and post-HFU mortality data are unpaired and categorical, we used Fischer's exact test for this purpose. Subsequently, we compared the conclusions drawn from these basic statistical tests with those drawn using our proposed modelling technique, to assess the potential benefits of the technique.

## 3. Results

**Figure 1:**
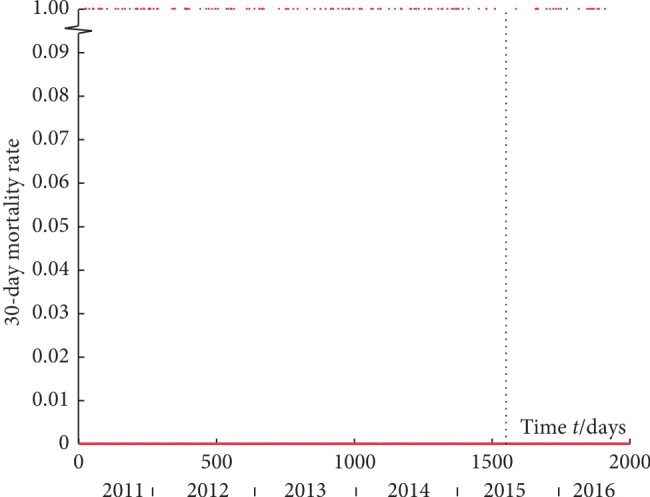
Scatter diagram of the time-series of 30-day mortality. Dashed vertical line is the onset of the HFU. Mortality data values are shown at either *y* = 0 or 1.

Scatter diagrams of binary event series are much less informative when compared to scatter diagrams of continuous variables when investigating time-dependent trends. Expectedly, with data values being grouped at *y* = 1 and *y* = 0, a scatter diagram of patient mortality does not offer any discernible information.  Figure 1 is a scatter diagram for 30-day mortality.

**Figure 2:**
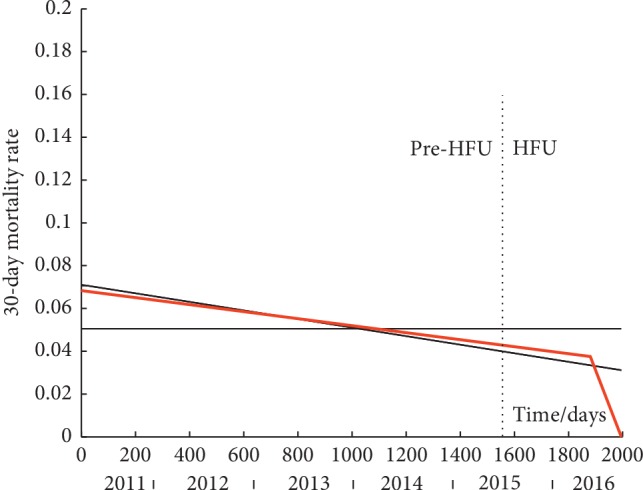
Modelling of the time-series of 30-day mortality. Solid red line is the best model. Solid black lines are the other models. Dashed vertical line is the onset of the HFU. Data values are not shown as they are points at either *y* = 0 or 1. (i) Plateau model ${\hat{y}}_{i}$ = 0.0505. (ii) Line model $\hat{y}$ = −0.000020*t* + 0.0711. (iii) Plateau-line model $\hat{y}$ = 0.0706 for *t* = 0 to 25.1 days and $\hat{y}$ = −0.000020 (*t* − 25.1) + 0.0706 for *t* = 25.1 to 1995 days. (iv) Line-line model $\hat{y}$ = −0.000016*t* + 0.0683 for *t* = 0 to 1880.4 days and $\hat{y}$ = −0.000328 (*t* − 1880.4) + 0.0375 for *t* = 1880.4 to 1995 days.

**Figure 3:**
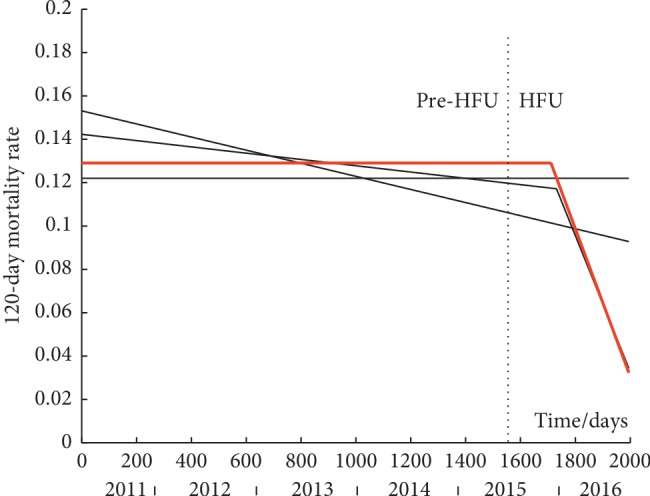
Modelling of the time-series of 120-day mortality. Solid red line is the best model. Solid black lines are the other models. Dashed vertical line is the onset of the HFU. Data values are not shown as they are points at either *y* = 0 or 1. (i) Plateau model $\hat{y}$ = 0.1221. (ii) Line model $\hat{y}$ = −0.000030*t* + 0.1531. (iii) Plateau-line model $\hat{y}$ = 0.1291 for *t* = 0 to 1358.1 days and $\hat{y}$ = −0.000341*t* + 0.1291 for *t* = 1358.1 to 1995 days. (iv) Line-line model $\hat{y}$ = −0.000015*t* + 0.1423 for *t* = 0 to 1731.2 days and $\hat{y}$ = −0.000328 (*t* − 1731.2) + 0.1172 for *t* = 1731.2 to 1995 days.

**Figure 4:**
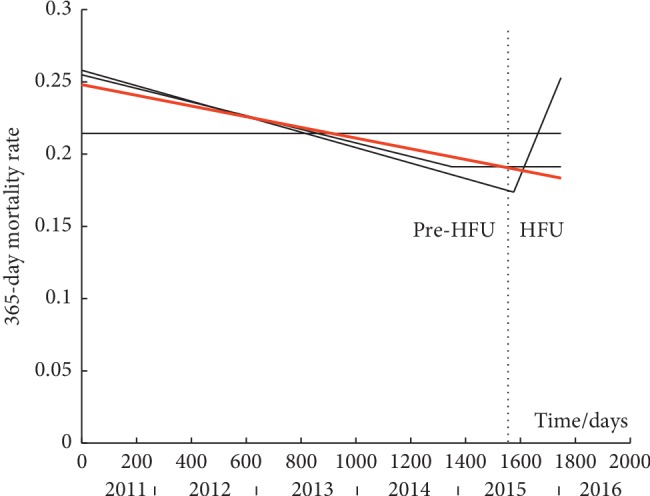
Modelling of the time-series of 365-day mortality. Solid red line is the best model. Solid black lines are the other models. Dashed vertical line is the onset of the HFU. Data values are not shown as they are points at either *y* = 0 or 1. (i) Plateau model $\hat{y}$ = 0.2144. (ii) Line model $\hat{y}$ = −0.000037*t* + 0.2481. (iii) Line-plateau model $\hat{y}$ = −0.000047*t* + 0.2549 for *t* = 0 to 1711 days and $\hat{y}$ = 0.1912 for *t* = 1711 to 1747.4 days (iv) Line-line model $\hat{y}$ = −0.000054*t* + 0.2580 for *t* = 0 to 1583.3 days and $\hat{y}$ = 0.000460 (*t* − 1583.3) + 0.1834 for *t* = 1583.3 to 1747.4 days.

By fitting the set of four piecewise linear models to each time-series, it is possible to discern trends. These are shown in Figures 2–4 for 30-day, 120-day, and 365-day mortality respectively. Data values are not shown as they are points at either *y* = 0 or 1. The best model for each time-series, as designated using AIC, is shown by a solid red line while the other three models (in black solid lines) are superimposed on the graphs for comparison. A dashed vertical line depicts the point of the intervention (introduction of the HFU).

### 3.1. 30-Day Mortality

Using Fischer's exact test, we found a significant reduction in average 30-day mortality from 5.47% pre-HFU to 3.13% post-HFU (*p*=0.014).

The best model to describe the time-series is the line-line (iv) model (Figure 1). Plateau is 0.043 as likely, line is 0.793 as likely, and plateau-line is 0.293 as likely. The plateau-line model is indistinguishable from the line model in the figure as its plateau section occupies a very brief initial phase only.

### 3.2. 120-Day Mortality

Using Fischer's exact test, we found a non-significant drop in 120-day mortality from 12.68% pre-HFU to 10.13% post-HFU (*p*=0.078).

The best model to describe the time-series is the plateau-line (iii) model. Plateau is 0.013 as likely, line is 0.2144 as likely, and line-line is 0.7011 as likely.

### 3.3. 365-Day Mortality

Using Fischer's exact test, we found a small and non-significant reduction in 365-day mortality from 21.46% pre-HFU to 20.57% post-HFU (*p*=0.769).

The best model to describe the time-series is the line (ii) model. Plateau is 0.185 as likely, line-plateau is 0.6269 as likely, and line-line is 0.7098 as likely.

## 4. Discussion

Using a novel technique for modelling binary variables in retrospective time-series, this study demonstrates the advantage of piecewise linear sections in conveying meaningful information. We employed the presented technique to model change in hip fracture patient outcomes to evaluate the effectiveness of introducing a dedicated HFU.

### 4.1. Model Application to the HFU Study

Following pre- and post-intervention group comparison, we inferred that there was a significant reduction in average 30-day mortality from 5.47% pre-HFU to 3.13% post-HFU (*p*=0.014). However, applying our modelling technique and finding that the line-line is the best model suggest that 30-day mortality declined at a rate of 0.06% per month from 6.8% at the onset of the study and then accelerated to a rate of decline of 1% per month after the 1730^th^ day reaching a near-zero value at the end of the study period. The models help explain that the difference found by group comparison via statistical testing was not an immediate consequence of the HFU but the result of a gradual decline which was nonetheless accelerated about a year after the HFU. Bearing in mind that models are not necessarily exclusive, the single line model is a close second-best model. It can therefore be concluded that 30-day mortality did not stay unchanged (*p*=0.041) but decreased over the entire study period. Its decrease seems to have accelerated (but this does not reach significance) after about a year following the onset of the HFU.

Pre- and postintervention group comparison inferred that there was a nonsignificant drop in 120-day mortality from 12.68% pre-HFU to 10.13% post-HFU (*p*=0.078). The plateau-line is the best model, and this supports the effectiveness of the HFU in reducing 120-day mortality especially as the knot (joining point) is shortly after the onset of the HFU. Although group comparison found a nonsignificant change, the likelihood of 120-day mortality to be a mere plateau is very small (*p*=0.013). It can therefore be concluded that the HFU caused a significant but gradual reduction in 120-day mortality which started to appear about six months after the onset of the HFU.

Pre- and postintervention group comparison inferred that there was a small and nonsignificant reduction of average 365-day mortality from 21.46% pre-HFU to 20.57% post-HFU (*p*=0.769). Among the four models, the line was deemed the best model. As relative likelihoods are rather large, this does not designate any of the other models as significantly worst. It is therefore impossible to exclude the possibility that 365-day mortality remained unchanged. Bearing in mind that many patients sustaining proximal femoral fragility fractures are frail with an already elevated preinjury mortality rate, it may not be surprising that 365-day mortality is less influenced by improvement in hip fracture management when compared to shorter-term survival.

### 4.2. Evaluation of the Modelling Technique

The current study demonstrates how temporal analysis using the proposed modelling method can elucidate the outcomes of group comparisons which are known to be unreliable especially when the data spans a long period. Importantly, by modelling the entire time-series without bias toward the point of intervention, the proposed modelling method offers an unbiased picture of the temporal evolution of the outcome measures and provides a valuable tool in the retrospective assessment of interventions. It allows delayed or anticipatory effects that may be connected to the intervention to be revealed without extra computation [8]. Modelling the binary variable of interest directly, as opposed to modelling a transformed variable as in logistic regression, improves the interpretability of the results.

We have previously published the use of segmented linear regression and demonstrated its application to hip fracture patient outcomes [5]. Although ITS is the considered the gold standard for evaluating the effectiveness of interventions in retrospective time-series, unlike our technique, it does not allow multiple linear segments to describe the time-series and is biased to detecting change at the point of the intervention [5].

In this study, we developed the segmented modelling technique further and tailored it to binary variables by using MLE. This exhibits the following advantages:

1. Using linear regression, it is possible that the best-fit lines will predict unrealistic values of greater than 1 or smaller than 0. By using MLE, we avoid this possibility.
2. When using F-tests it is necessary to ensure normality of residuals, though this is impossible when studying binary variables. By using AIC instead of F-tests, we overcome this hurdle.
3. Previously, we employed F-tests to determine the best model that was significantly better than any other model. However, it is pragmatic to conclude that more than one model may be a good descriptor of the time-series. The technique presented in this paper allows us to exclude unlikely models (*p* < 0.05) when compared to the best model but not immediately reject other models with *p* > 0.05. Consequentially, we can deduce a relative likelihood for each acceptable (*p* > 0.05) model compared to the best model. This provides researchers with more information and reflects the possibility that one model is not necessarily the only plausible description of the time-series.

### 4.3. Limitations

The set of proposed models are limited to two adjoining linear segments and as such may be unable to track more complex change over a long time-period. To address this, the method could be extended to include more adjoining linear segments, but this needs to be undertaken with caution to prevent overcomplicating a simple and meaningful approach to modelling trends.

Second, the method does not always yield certain “yes/no” answers for determining the effectiveness of an intervention; more than one model can be deemed “acceptable” (*p* > 0.05) when compared to the best model. However, given the plethora of possibilities of change and trends before and after interventions, our method is not intended to always yield a clear answer and represents a pragmatic, informative tool for researchers investigating retrospective time-series.

Finally, application of the method requires some dedicated programming as most statistical packages do not allow users to fit more than one linear section.

### 4.4. Future Work

The proposed sequence of models ranges from a single plateau to more complex forms, including a twin line that can track more complex temporal change. The method can be extended to include higher order models with three of more segments to accommodate yet more complex change. Moreover, disjointed segments can be allowed to model sudden change [4, 18]. Adaptations such as including autoregressive terms to account for periodicity can also be implemented to model events that exhibit seasonal variation. In this case, the periodic component of variation should be subtracted from the model in order to allow for the detection of other (non-seasonal) change which may be attributed to an intervention [19].

## 5. Conclusion

The proposed segmented linear regression modelling technique can be used to detect trends in time-series of binary variables in retrospective studies. This can be used to evaluate the effectiveness of healthcare interventions and to highlight secular trends.

## Data Availability

The data used to support the findings of this study are available from the corresponding author upon request.
